# The Impacts of Heatwaves on Mortality Differ with Different Study Periods: A Multi-City Time Series Investigation

**DOI:** 10.1371/journal.pone.0134233

**Published:** 2015-07-28

**Authors:** Xiao Yu Wang, Yuming Guo, Gerry FitzGerald, Peter Aitken, Vivienne Tippett, Dong Chen, Xiaoming Wang, Shilu Tong

**Affiliations:** 1 School of Public Health and Social Work, Institute of Health and Biomedical Innovation, Queensland University of Technology, Brisbane, Queensland, Australia; 2 School of Population Health, Faculty of Medicine and Biomedical Sciences, The University of Queensland, Brisbane, Queensland, Australia; 3 School of Public Health, Tropical Medicine and Rehabilitation Sciences, James Cook University, Townsville, Queensland, Australia; 4 School of Clinical Sciences, Institute of Health and Biomedical Innovation, Queensland University of Technology, Brisbane, Queensland, Australia; 5 Ecosystem Sciences, CSIRO, Melbourne, Victoria, Australia; 6 Climate Adaptation and Sustainable Development, CSIRO, Melbourne, Victoria, Australia; Columbia University, UNITED STATES

## Abstract

**Background:**

Different locations and study periods were used in the assessment of the relationships between heatwaves and mortality. However, little is known about the comparability and consistency of the previous effect estimates in the literature. This study assessed the heatwave—mortality relationship using different study periods in the three largest Australian cities (Brisbane, Melbourne and Sydney).

**Methods:**

Daily data on climatic variables and mortality for the three cities were obtained from relevant government agencies between 1988 and 2011. A consistent definition of heatwaves was used for these cities. Poisson generalised additive model was fitted to assess the impact of heatwaves on mortality.

**Results:**

Non-accidental and circulatory mortality significantly increased during heatwaves across the three cities even with different heatwave definitions and study periods. Using the summer data resulted in the largest increase in effect estimates compared to those using the warm season or the whole year data.

**Conclusion:**

The findings may have implications for developing standard approaches to evaluating the heatwave-mortality relationship and advancing heat health warning systems. It also provides an impetus to methodological advance for assessing climate change-related health consequences.

## Introduction

Heatwaves can cause a remarkable increase in mortality and morbidity, which is observed by a number of studies in different countries of the world [1–9]. It is projected that the frequency, intensity, duration and geographic extent of heatwaves will increase as climate change proceeds [10]. Thus, it is imperative to quantify the health impact of heatwaves and assess the disease burden attributable to climate change.

A heatwave is usually defined as two or more consecutive days with temperature above a certain temperature cut-off (e.g., 95^th^ centile) for a specific study period [2–5,11]. In previous studies, different study periods were used in the assessment of the relationship between heatwaves and mortality [3,4,12–16]. For example, some studies chose five months (i.e., May—September) to represent the warm season in Northern Hemisphere as the study period [3,4], while others used three months (June—August) [12,13]. Other different periods (e.g., 6 months and/or a whole year) were also used [14–16]. However, little is known about the comparability of the previous effect estimates identified in the literature.

This study compared the heatwave—mortality relationship using different study periods, and explored the similarities and differences in the assessment of the health impacts of heatwaves by using summer, warm season and the whole year data across different cities in Australia.

## Materials and Methods

This study included the three largest metropolitan cities in Australia—Brisbane, Melbourne and Sydney, which are the capital cities of Queensland, Victoria and New South Wales, respectively. Approximately half Australian population live in these cities [17]. In this study, we focused on three different study periods of data: summer (Dec–Feb), warm season (Nov–Mar) and the whole year during 1988–2011.

### Data collection

Daily climatic data on maximum temperature (MaxT) (°C) and minimum temperature (MinT) (°C) for these three cities during the period 1988–2011, relative humidity (%) for Brisbane (1988–2011), Melbourne and Sydney (Jan. 1988 to May 2009) were acquired from the Australian Bureau of Meteorology. We selected all available meteorological stations located within ≤30 km of each city’s Central Business District (CBD) (7 stations in Brisbane, 7 stations in Melbourne and 11 stations in Sydney) and the same sets of meteorological data were used in our previous study [11].

Daily data on non-accidental and circulatory mortality in these cities for the same period were obtained from the Australian Bureau of Statistics. These data were aggregated and no individual information was provided due to the reasons of privacy protection.

### Data analysis

Daily mean temperatures (MeanT) (°C) which averaged the values of daily maximum and minimum temperatures were used to investigate the effect of heatwaves on mortality in these cities, because our previous research shows that mean temperature was a slightly better predictor of mortality than other temperature indices [11,18]. A heatwave was defined as the mean temperature above a certain percentile (e.g., 90^th^, 95^th^, 98^th^ and 99^th^ centiles of mean temperature) for two or more consecutive days in the summer, the warm season and the whole year according to each city climatic conditions during 1988–2011. The heatwaves were coded as a binary variable of 1 or 0 on each day (i.e., 1 for the heatwave days while 0 for non-heatwave days). Poisson generalised additive model (GAM) was used to examine the heatwave effects on mortality for each city. Cumulative lagged effects of 0–3 days were assessed, as our previous work showed that the effects of heatwaves were acute and were unlikely to last for over 3 days [11]. We adjusted for an array of confounders in the model, including humidity, population size, day of week, trend and seasonality. We used natural cubic spline for humidity (df = 3) and day of the year or seasons (df = 4). Relative risks (RRs) and 95% confidence intervals (CIs) were calculated using the GAM model. For fitting the time series GAM, we used the ‘mgcv’ package in R software (V.2.14.1).

### Ethics Statement

Ethical approval was granted by Queensland University of Technology Human Research Ethics Committee. All patient records were anonymized and de-identified prior to analysis.

## Results

Table 1 shows the summary statistics on the climatic variables for the three cities. The highest and lowest temperatures (MaxT and MinT) were all observed in Melbourne for three study periods. However, the means of these temperature measures were the highest in Brisbane and the lowest in Melbourne. The means of humidity in Brisbane and Sydney were slightly higher in summer than other two periods while Melbourne was opposite.

**Table 1:** Summary statistics of climatic variables for the three Australian cities (1988–2011).

	Summer^a^				Warm season^b^				Whole year			
	Mean	SD	Min	Max	Mean	SD	Min	Max	Mean	SD	Min	Max
**Brisbane**												
MaxT (°C)	29.2	2.3	20.1	40.1	28.7	2.4	20.0	40.1	25.7	3.7	12.3	40.1
MinT (°C)	20.3	2.2	11.9	27.1	19.5	2.4	8.5	27.1	15.2	5.0	1.1	27.1
MeanT (°C)	24.8	1.9	18.3	33.6	24.1	2.1	16.6	33.6	20.5	4.1	9.7	33.6
Humidity (%)	70.5	8.7	30.2	96.8	70.2	8.9	17.9	96.8	69.2	10.9	17.2	97.4
**Melbourne**												
MaxT (°C)	25.6	5.8	13.9	46.7	24.6	5.7	12.5	46.7	20.0	6.1	7.9	46.7
MinT (°C)	13.8	3.3	5.8	27.6	13.1	3.4	2.8	27.6	10.0	4.2	-2.0	27.6
MeanT (°C)	19.7	3.9	10.9	35.5	18.9	3.9	9.2	35.5	15.0	4.8	4.6	35.5
Humidity (%)^**c**^	65.0	11.6	19.7	95.6	65.8	11.3	19.7	95.6	70.4	11.6	19.7	97.3
**Sydney**												
MaxT (°C)	27.1	4.1	16.6	44.0	26.3	4.1	12.5	44.0	22.8	4.9	10.3	44.0
MinT (°C)	18.0	2.4	9.8	26.1	17.2	2.7	7.7	26.1	13.0	4.8	0.5	26.1
MeanT (°C)	22.5	2.7	14.6	33.2	21.7	2.9	12.0	33.2	17.9	4.5	6.8	33.2
Humidity (%)^**c**^	70.0	11.0	28.1	98.1	69.9	11.1	26.6	98.1	68.3	12.8	23.9	98.1

^a^ December–February

^b^ November–March

^**c**^These data were only available from 1st Jan. 1988 to 31st May 2009.

Table 2 indicates the number of heatwave days for each city. A consistent definition of heatwaves was used in this study (i.e., two or more consecutive days with mean temperature above 90^th^, 95^th^, 98^th^ and 99^th^ centile). Brisbane had more heatwave days than Melbourne and Sydney across all heatwave definitions during three study periods.

**Table 2:** Heatwave days by different consecutive days and percentiles of mean temperature for the three Australian cities (1988–2011).

Percentile	Brisbane				Melbourne				Sydney			
(Mean T)	°C^c^	2day+	3day+	4day+	°C^c^	2day+	3day+	4day+	°C^c^	2day+	3day+	4day+
**Summer** ^**a**^												
99%	29.5	11	9	0	30.1	4	0	0	29.9	2	0	0
98%	28.6	25	17	8	28.9	21	9	0	28.8	9	3	0
95%	27.9	78	60	36	27.2	58	28	4	27.4	46	20	11
90%	27.2	164	112	79	25.3	140	70	22	26.2	112	68	47
**Warm season** ^**b**^												
99%	29.0	19	9	0	29.3	17	3	0	29.3	4	0	0
98%	28.3	47	31	19	28.3	31	9	0	28.2	19	9	6
95%	27.6	106	84	51	26.3	100	46	13	26.8	77	33	24
90%	26.7	296	206	155	24.4	230	126	78	25.6	218	124	76
**Whole year**												
99%	28.1	66	46	28	28.0	38	12	0	27.9	24	10	7
98%	27.6	106	84	51	26.4	98	46	13	26.9	68	32	20
95%	26.5	358	278	209	23.9	294	156	102	25.3	271	155	89
90%	25.5	800	672	564	21.5	713	489	333	23.8	704	502	358

^a^ December–February

^b^ November–March

^c^ Cut-off points of mean temperature (°C).

Table 3 shows the daily number of non-accidental and circulatory deaths in the three cities during the different study periods. Overall, the daily maximum number of deaths (including both non-accidental and circulatory diseases) was all recorded in the summer across these three cities. About half non-accidental deaths were caused by circulatory diseases. The elderly (aged 75 and over) deaths accounted for about 60%–63% and 67%–74% of all non-accidental and circulatory deaths, respectively, for the three cities. The ratio of male to female deaths was the same (i.e., 1:1) across these cities.

**Table 3:** Daily numbers of non-accidental and circulatory deaths for the three Australian cities (1988–2011).

	Summer^a^				Warm season^b^				Whole year			
	Mean	SD	Min	Max	Mean	SD	Min	Max	Mean	SD	Min	Max
**Non-accidental deaths**												
**Brisbane**												
Male	12	4	2	38	12	4	2	38	13	4	1	38
Female	12	4	1	40	12	4	1	40	13	4	1	40
0–74	10	4	1	25	10	4	1	27	10	4	1	27
75+	15	5	2	46	15	5	2	46	16	5	1	46
Total	24	6	4	68	24	6	4	68	26	6	4	68
**Melbourne**												
Male	27	6	11	52	27	6	10	52	29	6	6	58
Female	27	6	10	79	27	6	10	79	29	6	7	79
0–74	21	6	3	49	21	6	3	49	22	6	1	49
75+	33	8	11	91	33	8	11	91	36	8	11	91
Total	54	9	23	127	54	8	23	127	58	9	13	127
**Sydney**												
Male	32	7	6	74	32	7	6	75	35	8	6	75
Female	31	7	9	70	31	7	6	70	34	8	6	70
0–74	26	8	6	69	26	8	6	74	27	8	3	74
75+	37	8	10	70	37	8	9	70	42	10	9	87
Total	62	11	20	136	62	11	20	136	69	13	20	136
**Circulatory deaths**												
**Brisbane**												
Male	6	2	0	18	6	2	0	18	7	2	0	18
Female	6	2	0	24	6	2	0	24	7	2	0	24
0–74	4	3	0	12	4	3	0	12	5	3	0	13
75+	8	2	0	33	8	2	0	33	9	3	0	33
Total	12	3	3	38	12	3	3	38	13	4	3	38
**Melbourne**												
Male	10	3	3	27	10	3	3	27	11	3	3	28
Female	11	3	3	41	11	3	3	41	12	4	3	41
0–74	7	2	0	18	7	2	0	21	8	3	0	21
75+	15	4	5	53	15	4	5	53	17	5	5	53
Total	21	5	7	67	21	5	7	67	23	6	7	67
**Sydney**												
Male	12	4	5	37	12	4	5	37	13	5	5	37
Female	13	4	5	37	13	4	0	37	15	5	0	43
0–74	8	3	0	27	8	3	0	27	9	3	0	27
75+	17	4	6	35	18	5	5	35	21	6	5	53
Total	25	6	9	64	25	7	8	64	29	8	8	66

^a^ December–February

^b^ November–March.

Table 4 depicts the relative risk (RR) of the non-accidental mortality in the three cities after adjustment for confounding factors in the model. Regardless of which heatwave definition was used, there was a statistically significant increase in mortality for almost all subgroups across three cities during heatwaves, particularly when the summer was used as the study period. In general, women were affected more by heat effects than men while the elderly (i.e., 75 years old or over) seemed more vulnerable to heatwaves than others, although not all RRs were statistically significant. Generally, the more intense the heatwave, the higher the RRs for non-accidental deaths.

**Table 4:** Relative risk (RR) of non-accidental mortality during heatwaves in the three Australian cities, 1988–2011.

	Mean	RR [95%CI]*		
	Temperature	Summer^a^	Warm season^b^	Whole year
**Brisbane**				
Total	99%	**1.40 [1.26–1.55]**	**1.30 [1.20–1.41]**	**1.14 [1.09–1.20]**
	98%	**1.32 [1.23–1.42]**	**1.18 [1.12–1.25]**	**1.10 [1.05–1.14]**
	95%	**1.08 [1.04–1.14]**	**1.09 [1.05–1.13]**	**1.05 [1.03–1.08]**
	90%	**1.06 [1.02–1.09]**	**1.06 [1.03–1.09]**	**1.06 [1.04–1.08]**
Male	99%	**1.22 [1.05–1.42]**	1.11 [0.98–1.25]	**1.08 [1.01–1.15]**
	98%	**1.17 [1.05–1.30]**	1.07 [0.99–1.16]	1.04 [0.99–1.10]
	95%	1.02 [0.96–1.09]	1.04 [0.98–1.10]	1.02 [0.98–1.05]
	90%	1.01 [0.96–1.06]	1.02 [0.99–1.06]	**1.05 [1.02–1.07]**
Female	99%	**1.56 [1.36–1.79]**	**1.48 [1.33–1.65]**	**1.20 [1.12–1.28]**
	98%	**1.46 [1.32–1.61]**	**1.27 [1.18–1.37]**	**1.13 [1.07–1.20]**
	95%	**1.14 [1.07–1.21]**	**1.13 [1.07–1.19]**	**1.08 [1.04–1.11]**
	90%	**1.09 [1.04–1.14]**	**1.08 [1.04–1.12]**	**1.07 [1.05–1.10]**
0–74	99%	**1.28 [1.08–1.51]**	**1.19 [1.04–1.36]**	1.04 [0.96–1.12]
	98%	**1.19 [1.06–1.34]**	1.07 [0.98–1.17]	1.03 [0.97–1.10]
	95%	1.02 [0.95–1.10]	1.03 [0.96–1.10]	1.01 [0.97–1.05]
	90%	0.99 [0.94–1.05]	1.02 [0.97–1.06]	**1.04 [1.01–1.07]**
75+	99%	**1.46 [1.28–1.66]**	**1.36 [1.23–1.50]**	**1.20 [1.13–1.27]**
	98%	**1.39 [1.27–1.52]**	**1.24 [1.15–1.33]**	**1.12 [1.07–1.18]**
	95%	**1.11 [1.05–1.18]**	**1.12 [1.06–1.17]**	**1.08 [1.05–1.11]**
	90%	**1.09 [1.05–1.14]**	**1.08 [1.05–1.12]**	**1.08 [1.06–1.11]**
**Melbourne**				
Total	99%	**1.44 [1.29–1.62]**	**1.12 [1.04–1.19]**	**1.08 [1.03–1.13]**
	98%	**1.09 [1.02–1.16]**	**1.06 [1.01–1.11]**	**1.04 [1.01–1.07]**
	95%	**1.04 [1.00–1.08]**	**1.03 [1.00–1.06]**	**1.03 [1.01–1.05]**
	90%	**1.04 [1.01–1.07]**	**1.04 [1.01–1.06]**	**1.02 [1.00–1.03]**
Male	99%	**1.33 [1.13–1.58]**	1.06 [0.96–1.17]	1.01 [0.95–1.08]
	98%	1.02 [0.93–1.12]	0.99 [0.92–1.06]	1.00 [0.96–1.04]
	95%	0.99 [0.94–1.05]	1.00 [0.95–1.04]	1.01 [0.98–1.04]
	90%	1.02 [0.98–1.06]	1.01 [0.98–1.05]	1.01 [0.99–1.03]
Female	99%	**1.55 [1.33–1.82]**	**1.17 [1.07–1.28]**	**1.15 [1.08–1.22]**
	98%	**1.16 [1.06–1.26]**	**1.13 [1.06–1.22]**	**1.07 [1.03–1.12]**
	95%	**1.08 [1.02–1.14]**	**1.07 [1.03–1.12]**	**1.06 [1.03–1.09]**
	90%	**1.06 [1.01–1.10]**	**1.06 [1.03–1.09]**	**1.02 [1.01–1.04]**
0–74	99%	**1.27 [1.04–1.56]**	1.01 [0.90–1.14]	0.98 [0.91–1.06]
	98%	0.97 [0.87–1.08]	1.01 [0.93–1.10]	0.99 [0.95–1.04]
	95%	0.96 [0.90–1.02]	1.00 [0.95–1.05]	1.01 [0.98–1.04]
	90%	1.01 [0.96–1.06]	1.02 [0.99–1.06]	1.01 [0.99–1.03]
75+	99%	**1.56 [1.36–1.80]**	**1.18 [1.09–1.28]**	**1.15 [1.09–1.21]**
	98%	**1.17 [1.08–1.26]**	**1.09 [1.03–1.16]**	**1.06 [1.02–1.10]**
	95%	**1.09 [1.04–1.14]**	**1.05 [1.01–1.10]**	**1.05 [1.02–1.07]**
	90%	**1.06 [1.02–1.09]**	**1.05 [1.02–1.08]**	**1.02 [1.00–1.04]**
**Sydney**				
Total	99%	NA	NA	**1.12 [1.05–1.19]**
	98%	**1.32 [1.13–1.55]**	**1.14 [1.06–1.22]**	**1.06 [1.02–1.09]**
	95%	**1.06 [1.01–1.10]**	**1.07 [1.04–1.10]**	**1.04 [1.02–1.06]**
	90%	**1.05 [1.02–1.08]**	**1.05 [1.03–1.07]**	**1.03 [1.02–1.04]**
Male	99%	NA^c^	NA	**1.11 [1.02–1.21]**
	98%	**1.52 [1.24–1.87]**	**1.15 [1.04–1.27]**	**1.04 [1.00–1.09]**
	95%	1.05 [0.99–1.11]	**1.07 [1.02–1.11]**	1.01 [0.98–1.03]
	90%	**1.04 [1.00–1.08]**	1.02 [0.99–1.05]	1.01 [0.99–1.02]
Female	99%	NA	NA	**1.13 [1.03–1.23]**
	98%	1.15 [0.91–1.45]	**1.14 [1.02–1.26]**	**1.06 [1.01–1.11]**
	95%	1.05 [0.98–1.12]	**1.06 [1.02–1.11]**	**1.06 [1.04–1.09]**
	90%	**1.06 [1.02–1.10]**	**1.06 [1.04–1.09]**	**1.05 [1.03–1.07]**
0–74	99%	NA	NA	**1.12 [1.02–1.23]**
	98%	1.18 [0.93–1.51]	1.09 [0.98–1.23]	1.03 [0.97–1.08]
	95%	1.02 [0.96–1.10]	1.04 [0.99–1.09]	**1.02 [1.00–1.05]**
	90%	1.01 [0.97–1.06]	**1.03 [1.00–1.06]**	**1.03 [1.01–1.05]**
75+	99%	NA	NA	**1.12 [1.04–1.21]**
	98%	**1.47 [1.20–1.80]**	**1.18 [1.07–1.29]**	**1.08 [1.03–1.12]**
	95%	**1.07 [1.02–1.14]**	**1.09 [1.05–1.13]**	**1.04 [1.02–1.07]**
	90%	**1.08 [1.04–1.12]**	**1.05 [1.02–1.08]**	**1.03 [1.01–1.04]**

*Adjusted confounders including humidity, day of week, day of year, population size and season for whole year

^a^ December–February

^b^ November–March

^c^ Heatwaves occurred in Jan 2010 and Feb 2011 in Sydney but humidity data were unavailable

Bold typeface indicates statistical significance at p<0.

The RRs for daily circulatory mortality during heatwaves using the different heatwave definitions and different study periods in the three cities before and after adjusting for confounders were shown in S1 Table. In general, heatwaves appeared to have greater impacts on circulatory mortality than non-accidental mortality in these cities (Table 4 and S1 Table). The patterns of susceptibility is similar to that observed in non-accidental deaths–viz., in most cases, females and the elderly aged 75 years or over appeared to be more vulnerable to heatwaves than males and the non-elderly, regardless of which definition was used.

Table 5 shows the relative risk of mortality in single lag (lag 1 to lag 3) and cumulative lag (Cum lag 0–3) effects during heatwaves defined as two or more consecutive days above a certain percentile of daily mean temperature using either the summer or whole year data. There was a consistent, immediate increase in mortality during heatwaves for almost all heatwave definitions across the three cities.

**Table 5:** Relative risk (RR) of non-accidental mortality in lag effects during heatwaves in the three Australian cities, 1988–2011.

Percentile of Mean Temperature	RR [95%CI]*		
	Brisbane	Melbourne	Sydney
Summer			
Lag 1			
99%	**1.46 [1.32–1.61]**	**1.76 [1.59–1.95]**	NA
98%	**1.31 [1.21–1.41]**	**1.20 [1.13–1.27]**	**1.24 [1.05–1.46]**
95%	**1.10 [1.05–1.15]**	**1.08 [1.05–1.12]**	**1.07 [1.02–1.11]**
90%	**1.03 [1.00–1.07]**	**1.05 [1.03–1.08]**	**1.02 [1.00–1.05]**
Lag 2			
99%	**1.46 [1.32–1.63]**	**1.45 [1.29–1.62]**	NA
98%	**1.28 [1.19–1.38]**	**1.18 [1.11–1.25]**	1.16 [0.99–1.37]
95%	**1.09 [1.04–1.14]**	**1.07 [1.03–1.11]**	**1.04 [1.00–1.09]**
90%	**1.03 [1.00–1.07]**	**1.04 [1.01–1.06]**	**1.02 [1.00–1.05]**
Lag 3			
99%	**1.20 [1.06–1.36]**	**1.16 [1.02–1.31]**	NA
98%	**1.29 [1.19–1.40]**	**1.09 [1.02–1.15]**	**1.21 [1.03–1.42]**
95%	**1.07 [1.02–1.12]**	**1.04 [1.00–1.08]**	**1.02 [0.97–1.07]**
90%	**1.03 [1.00–1.07]**	**1.03 [1.00–1.05]**	1.01 [0.99–1.04]
Cumulative lag 0–3			
99%	**3.26 [2.40–4.42]**	**3.85 [2.87–5.17]**	NA
96%	**2.58 [2.07–3.20]**	**1.42 [1.21–1.67]**	**2.48 [1.65–3.72]**
95%	**1.21 [1.05–1.39]**	**1.16 [1.05–1.28]**	**1.18 [1.05–1.33]**
90%	1.08 [0.97–1.20]	**1.09 [1.01–1.17]**	**1.13 [1.05–1.22]**
Whole year			
Lag 1			
99%	**1.17 [1.11–1.22]**	**1.13 [1.08–1.18]**	**1.13 [1.06–1.20]**
98%	**1.10 [1.06–1.15]**	**1.06 [1.03–1.09]**	**1.05 [1.02–1.08]**
95%	**1.04 [1.01–1.06]**	**1.03 [1.01–1.05]**	**1.02 [1.00–1.03]**
90%	**1.05 [1.03–1.07]**	1.00 [0.99–1.02]	**1.02 [1.01–1.03]**
Lag 2			
99%	**1.15 [1.09–1.21]**	**1.11 [1.06–1.16]**	1.05 [0.98–1.11]
98%	**1.08 [1.04–1.12]**	**1.05 [1.02–1.08]**	**1.04 [1.01–1.08]**
95%	**1.03 [1.01–1.06]**	1.01 [0.99–1.03]	1.00 [0.98–1.02]
90%	**1.04 [1.02–1.06]**	0.99 [0.98–1.00]	1.00 [0.99–1.02]
Lag 3			
99%	**1.14 [1.08–1.20]**	**1.05 [1.01–1.10]**	1.04 [0.97–1.10]
98%	**1.07 [1.03–1.12]**	**1.03 [1.00–1.06]**	1.02 [0.99–1.06]
95%	**1.02 [1.00–1.05]**	1.01 [0.99–1.02]	0.99 [0.97–1.01]
90%	**1.03 [1.01–1.05]**	0.99 [0.98–1.00]	0.99 [0.98–1.00]
Cumulative lag 0–3			
99%	**1.54 [1.33–1.78]**	**1.28 [1.14–1.44]**	**1.34 [1.14–1.57]**
96%	**1.24 [1.10–1.41]**	**1.13 [1.05–1.23]**	**1.18 [1.07–1.29]**
95%	**1.18 [1.09–1.27]**	1.03 [0.97–1.08]	**1.06 [1.01–1.12]**
90%	**1.24 [1.16–1.32]**	0.97 [0.93–1.01]	**1.05 [1.01–1.09]**

*Adjusted confounders including humidity, day of week, day of year, population size and season for whole year

Bold typeface indicates statistical significance at p<0.05.

## Discussion

In this study, we analysed the associations between heatwaves and mortality using the data for different study periods in the three largest Australian cities (Brisbane, Melbourne and Sydney). The results show that, regardless of which heatwave definition was used, there were consistent, statistically significant, increased risks of mortality during heatwaves even using different study periods. It is more sensitive to use the summer data than the warm season or the whole year data in assessing heatwaves-related health risks but the magnitude of risks varied with city. Finally, we found a stronger effect of heatwaves on circulatory mortality than overall non-accidental mortality across all three cities.

Several multi-city studies have reported the mortality impacts of heatwaves [3–5,16,19]. However, in previous studies different study periods were used to assess the heatwave-mortality relationship. The findings of this study show that the effect estimates using the summer data were greater than those estimated by using either the warm season or the whole year data. It is due largely to the different intensity of heat categorized by different study periods even though the same percentiles of temperature were applied in the definition of a heatwave.

There were different climatic patterns across Brisbane, Melbourne and Sydney. Brisbane is close to the northern east coast and has a humid subtropical climate with warm to hot and humid summers, and dry and moderately warm winters [20]. Melbourne has a moderate oceanic climate and changeable weather conditions [21,22]. Sydney’s weather is between Brisbane and Melbourne which has a temperate climate with warm summers and mild winters, with rainfall spread throughout the year [23]. Even though these cities have different climatic conditions, consistent, statistically significant, increased risks of mortality were observed during heatwaves across all these cities. It suggests that residents living in different climates were all susceptible to extreme heat effects and up to now, the role of adaptation was limited when a heatwave occurred.

Clearly, most heatwave events occurred in summer. For example, when two or more consecutive days above the 95^th^ percentile of mean temperature were used to define a heatwave using the whole year data, there were 358 heatwave days in total and 330 (92%) days occurred in summer in Brisbane; similarly 81% and 86% heatwave days occurred in summer in Melbourne and Sydney, respectively. When the summer data were used, heatwaves appeared to have a greater impact on non-accidental mortality, elderly aged 75 years or over and females (Table 4 and S1 Table), which is consistent with previous studies [4,9,13,24,25]. Similar but less significant results were observed when the warm season and the whole year data were used.

We also investigated the lag effects of heatwaves in the summer and whole year, and found that the higher risk estimates for mortality were on the current day (lag 0) and lag of 1 day. Our results support the findings that the impact of heatwaves on mortality is usually acute and does not last long [15].

This study has three key strengths. To the best of our knowledge, this is the first study to compare the effect estimates using different study periods in examining the health impacts of heatwaves across different cities. Comprehensive datasets (e.g., 24 years) on population, meteorological conditions and mortality for these cities were used, and key confounding factors were adjusted for in the model. Finally, a consistent definition was applied to define a heatwave based on local climatic conditions with different study periods.

This study also has some limitations. The aggregated data on non-accidental and circulatory deaths were used while individual information on exposure, outcomes and confounders was unavailable. Different cutoffs (i.e., 90th, 95th 98th and 99th percentile) of mean temperature were used to define a heatwave, and multiple significant tests were conducted. Potential confounding effects of air pollution (e.g., ozone) were not controlled for, as these data were not complete for the whole study period in all three cities. However, there is evidence that the association between heat and mortality is likely to be independent of air pollution [15]. It is also debated whether air pollution should be adjusted for in the studies of temperature effects [26].

## Conclusions

It appears to be more sensitive to use the summer data rather than the warm season or the whole year data in assessing the heatwaves-mortality relationship. Regardless of which study period was used, a consistent and significant increase in mortality was observed during heatwaves across the three major Australian cities. The findings may have significant implications for developing standard approaches to evaluating the heatwave-mortality relationship and advancing heat health warning systems. It also provides an impetus to methodological advancement for assessing climate change-related health consequences.

## Supporting Information

S1 Table Relative risk (RR) of circulatory mortality during heatwaves in the three Australian cities (1988–2011). (DOCX)
