# Supplementary material for: The Impacts of Heatwaves on Mortality Differ with Different Study Periods: A Multi-City Time Series Investigation
Source: PLoS One. 2015 Jul 28;10(7):e0134233. doi: 10.1371/journal.pone.0134233 (PMC4517756; doi:10.1371/journal.pone.0134233)
Supplement: S1 Table — (DOCX) [file pone.0134233.s001.docx]

**S1 Table** Relative risk (RR) of circulatory mortality during heatwaves in the three Australian cities (1988 – 2011)

|  | Mean | RR [95%CI]* | | | | |
| --- | --- | --- | --- | --- | --- | --- |
|  | Temperature | Summer^a^ |  | Warm season^b^ |  | Whole year |
| **Brisbane** |  |  |  |  |  |  |
| total | 99% | **1.42 [1.22 - 1.66]** |  | **1.34 [1.17 - 1.53]** |  | **1.32 [1.21 - 1.44]** |
|  | 98% | **1.40 [1.25 - 1.57]** |  | **1.34 [1.22 - 1.48]** |  | **1.27 [1.17 - 1.37]** |
|  | 95% | **1.30 [1.20 - 1.42]** |  | **1.27 [1.17 - 1.37]** |  | **1.09 [1.04 - 1.15]** |
|  | 90% | **1.19 [1.11 - 1.27]** |  | **1.12 [1.06 - 1.18]** |  | **1.05 [1.01 - 1.09]** |
| male | 99% | **1.39 [1.09 - 1.76]** |  | **1.36 [1.11 - 1.65]** |  | **1.28 [1.14 - 1.43]** |
|  | 98% | **1.47 [1.25 - 1.73]** |  | **1.35 [1.18 - 1.54]** |  | **1.21 [1.10 - 1.33]** |
|  | 95% | **1.22 [1.10 - 1.35]** |  | **1.21 [1.10 - 1.33]** |  | 1.05 [0.99 - 1.12] |
|  | 90% | **1.11 [1.02 - 1.21]** |  | **1.06 [0.99 - 1.14]** |  | 1.03 [0.99 - 1.08] |
| female | 99% | **1.67 [1.36 - 2.05]** |  | **1.55 [1.31 - 1.84]** |  | **1.24 [1.12 - 1.38]** |
|  | 98% | **1.46 [1.26 - 1.69]** |  | **1.28 [1.13 - 1.43]** |  | **1.18 [1.08 - 1.28]** |
|  | 95% | **1.22 [1.10 - 1.35]** |  | **1.18 [1.08 - 1.28]** |  | **1.09 [1.03 - 1.15]** |
|  | 90% | **1.11 [1.02 - 1.19]** |  | **1.11 [1.04 - 1.18]** |  | **1.07 [1.03 - 1.12]** |
| 0-74 | 99% | 1.24 [0.88 - 1.75] |  | 1.25 [0.92 - 1.69] |  | **1.33 [1.08 - 1.63]** |
|  | 98% | 1.26 [0.99 - 1.61] |  | **1.29 [1.03 - 1.62]** |  | **1.23 [1.02 - 1.49]** |
|  | 95% | **1.30 [1.06 - 1.60]** |  | **1.24 [1.03 - 1.49]** |  | 0.97 [0.86 - 1.09] |
|  | 90% | 1.12 [0.95 - 1.32] |  | 1.02 [0.90 - 1.16] |  | 1.00 [0.92 - 1.09] |
| 75+ | 99% | **1.61 [1.36 - 1.91]** |  | **1.45 [1.26 - 1.67]** |  | **1.28 [1.18 - 1.39]** |
|  | 98% | **1.54 [1.37 - 1.74]** |  | **1.32 [1.20 - 1.45]** |  | **1.22 [1.14 - 1.30]** |
|  | 95% | **1.24 [1.15 - 1.34]** |  | **1.21 [1.13 - 1.29]** |  | **1.10 [1.05 - 1.15]** |
|  | 90% | **1.13 [1.07 - 1.20]** |  | **1.10 [1.05 - 1.15]** |  | **1.05 [1.01 - 1.08]** |
| **Melbourne** |  |  |  |  |  |  |
| total | 99% | **1.76 [1.48 - 2.10]** |  | **1.22 [1.10 - 1.37]** |  | **1.15 [1.07 - 1.23]** |
|  | 98% | **1.18 [1.06 - 1.31]** |  | **1.12 [1.03 - 1.21]** |  | **1.06 [1.01 - 1.11]** |
|  | 95% | **1.06 [1.00 - 1.14]** |  | 1.04 [0.99 - 1.10] |  | **1.05 [1.02 - 1.09]** |
|  | 90% | 1.04 [0.99 - 1.09] |  | **1.05 [1.01 - 1.09]** |  | **1.03 [1.01 - 1.05]** |
| male | 99% | **1.59 [1.20 - 2.09]** |  | 1.06 [0.90 - 1.25] |  | 0.98 [0.88 - 1.10] |
|  | 98% | 1.05 [0.90 - 1.23] |  | 0.98 [0.87 - 1.12] |  | 0.99 [0.92 - 1.06] |
|  | 95% | 0.98 [0.89 - 1.08] |  | 0.98 [0.91 - 1.06] |  | 0.99 [0.94 - 1.04] |
|  | 90% | 0.97 [0.91 - 1.04] |  | 0.99 [0.94 - 1.05] |  | 1.00 [0.97 - 1.03] |
| female | 99% | **1.89 [1.51 - 2.37]** |  | **1.33 [1.15 - 1.54]** |  | **1.29 [1.18 - 1.42]** |
|  | 98% | **1.30 [1.14 - 1.49]** |  | **1.24 [1.12 - 1.38]** |  | **1.15 [1.08 - 1.23]** |
|  | 95% | **1.14 [1.05 - 1.24]** |  | **1.13 [1.06 - 1.21]** |  | **1.12 [1.07 - 1.17]** |
|  | 90% | **1.10 [1.03 - 1.17]** |  | **1.10 [1.05 - 1.16]** |  | **1.07 [1.03 - 1.10]** |
| 0-74 | 99% | 1.00 [0.67 - 1.49] |  | 1.06 [0.83 - 1.34] |  | 1.06 [0.90 - 1.25] |
|  | 98% | 1.03 [0.82 - 1.29] |  | 1.06 [0.88 - 1.26] |  | 0.99 [0.89 - 1.10] |
|  | 95% | 0.95 [0.82 - 1.10] |  | 0.99 [0.88 - 1.10] |  | 1.01 [0.95 - 1.08] |
|  | 90% | 0.96 [0.87 - 1.06] |  | 1.01 [0.94 - 1.10] |  | 1.01 [0.97 - 1.06] |
| 75+ | 99% | **1.89 [1.55 - 2.29]** |  | **1.19 [1.05 - 1.34]** |  | **1.17 [1.08 - 1.27]** |
|  | 98% | **1.17 [1.05 - 1.31]** |  | **1.12 [1.02 - 1.23]** |  | **1.07 [1.01 - 1.13]** |
|  | 95% | **1.08 [1.00 - 1.16]** |  | 1.05 [0.99 - 1.11] |  | **1.06 [1.03 - 1.10]** |
|  | 90% | **1.05 [1.00 - 1.11]** |  | **1.04 [1.00 - 1.09]** |  | **1.03 [1.01 - 1.06]** |
| **Sydney** |  |  |  |  |  |  |
| total | 99% | NA^c^ |  | NA |  | **1.14 [1.04 - 1.26]** |
|  | 98% | **1.51 [1.21 - 1.89]** |  | **1.21 [1.09 - 1.35]** |  | **1.08 [1.02 - 1.13]** |
|  | 95% | **1.07 [1.00 - 1.14]** |  | **1.08 [1.03 - 1.14]** |  | **1.06 [1.03 - 1.08]** |
|  | 90% | **1.07 [1.02 - 1.11]** |  | **1.06 [1.03 - 1.09]** |  | **1.03 [1.01 - 1.05]** |
| male | 99% | NA |  | NA |  | 1.13 [0.99 - 1.30] |
|  | 98% | **1.85 [1.36 - 2.51]** |  | **1.24 [1.06 - 1.45]** |  | 1.02 [0.95 - 1.10] |
|  | 95% | 1.02 [0.93 - 1.13] |  | 1.05 [0.98 - 1.13] |  | 1.02 [0.99 - 1.07] |
|  | 90% | 1.02 [0.96 - 1.09] |  | 1.02 [0.98 - 1.07] |  | 1.01 [0.99 - 1.04] |
| female | 99% | NA |  | NA |  | **1.15 [1.01 - 1.31]** |
|  | 98% | 1.22 [0.88 - 1.69] |  | **1.19 [1.02 - 1.38]** |  | **1.13 [1.05 - 1.21]** |
|  | 95% | **1.10 [1.01 - 1.20]** |  | **1.11 [1.04 - 1.19]** |  | **1.09 [1.05 - 1.13]** |
|  | 90% | **1.10 [1.04 - 1.16]** |  | **1.10 [1.06 - 1.15]** |  | **1.04 [1.02 - 1.07]** |
| 0-74 | 99% | NA |  | NA |  | 1.13 [0.94 - 1.36] |
|  | 98% | **1.48 [1.00 - 2.20]** |  | 1.16 [0.94 - 1.42] |  | 0.95 [0.86 - 1.06] |
|  | 95% | 0.96 [0.84 - 1.09] |  | 0.97 [0.88 - 1.07] |  | 1.01 [0.96 - 1.06] |
|  | 90% | 0.98 [0.91 - 1.06] |  | 1.00 [0.94 - 1.06] |  | 1.02 [0.98 - 1.06] |
| 75+ | 99% | NA |  | NA |  | **1.15 [1.03 - 1.29]** |
|  | 98% | **1.46 [1.12 - 1.91]** |  | **1.18 [1.03 - 1.34]** |  | **1.14 [1.08 - 1.21]** |
|  | 95% | **1.11 [1.03 - 1.20]** |  | **1.13 [1.07 - 1.20]** |  | **1.08 [1.05 - 1.11]** |
|  | 90% | **1.12 [1.07 - 1.17]** |  | **1.08 [1.05 - 1.12]** |  | **1.04 [1.02 - 1.06]** |

*Adjusted confounders including humidity, day of week, day of year, population size and season for whole year;

^a^ December – February;

^b^ November – March;

^c^ Heatwaves occurred in Jan 2010 and Feb 2011 in Sydney while humidity data not available;

Bold typeface indicates statistical significance at p<0.
